# Outcomes from Experimental Testing of Nonsteroidal Anti-Inflammatory Drug (NSAID) Administration during the Transition Period of Dairy Cows

**DOI:** 10.3390/ani10101832

**Published:** 2020-10-08

**Authors:** Francesca Trimboli, Monica Ragusa, Cristian Piras, Vincenzo Lopreiato, Domenico Britti

**Affiliations:** 1 Department of Health Science, Magna Græcia University, 88100 Catanzaro, Italy; trimboli@unicz.it (F.T.); c.piras@reading.ac.uk (C.P.); britti@unicz.it (D.B.); 2 Department of Experimental and Clinical Medicine, Magna Græcia University, 88100 Catanzaro, Italy; m.ragusa@unicz.it; 3 Department of Animal Sciences, Food and Nutrition, Faculty of Agriculture, Food and Environmental Science, Università Cattolica del Sacro Cuore, 29122 Piacenza, Italy

**Keywords:** NSAID, transition period, milk production, cow health

## Abstract

**Simple Summary:**

The treatment of dairy cows with nonsteroidal drugs is applied experimentally to investigate the relevance of inflammation during the periparturient period. Despite appearing healthy, dairy cows throughout the transition period and mainly after parturition can develop a pro-inflammatory status that may negatively influence milk production and cows’ health. The administration of nonsteroidal anti-inflammatory drugs (NSAIDs) has been demonstrated to have both positive or negative effects on health and milk production, depending on the type of inhibition mechanism, the dose administered and the cows’ lactation numbers. At present, the safety and efficacy of NSAIDs have not been irrefutably demonstrated; therefore, their use to improve metabolic and inflammatory status, as well as milk production and cow health after parturition, should be carefully evaluated.

**Abstract:**

During the transition period, dairy cows experience great physiological stress caused by changes in metabolism and in the immune and endocrine systems. A pro-inflammatory state is another difficulty faced by even apparently healthy animals. The most significant negative consequences of inflammation in dairy cows are substantial impairment of milk production and deleterious effects on cows’ health in extreme cases. Nonetheless, a certain degree of inflammation is necessary to sustain physiological adaptations. In recent years, many studies have attempted to determine whether the use of nonsteroidal anti-inflammatory drugs (NSAID) in the transition period of dairy cows could positively affect milk production and cows’ health by controlling the inflammation status. This literature indicates that NSAIDs that act as preferential inhibitors of cyclooxygenase-1 (COX-1) activity show important side effects (e.g., increased risk of retained placenta, culling, or metritis) even if milk production is, on average, ameliorated. In contrast, preferential inhibitors of cyclooxygenase-2 (COX-2) activity have overall positive effects on cows’ health, with potential beneficial effects on milk production. Furthermore, it is important to note that with certain NSAID treatments, milk discarding is mandatory to prevent contamination with drug residues, but increased milk production can compensate for the loss of milk revenue during the withdrawal period.

## 1. Introduction

The transition period (TP), the most critical physiological stage in dairy cattle, starts 3 weeks before parturition and finishes 3 weeks thereafter. It is a critical physiological phase for dairy cattle due to the major changes that occur in metabolism, immune systems, and endocrine system [1]. During this period, dairy cows often experience a negative energy balance (NEB) and micronutrient deficiencies [2]. A NEB in transition cows is mainly caused by two phenomena: First, immediately after parturition, the energy requirements dairy cows rapidly increases due to enormous milk secretion; and second, there is a concurrent significant reduction in feed intake [3,4]. Lactogenesis induces an increase in body fat mobilization toward the mammary glands and a subsequent increase in serum NEFA and BHB levels [5]. Both hypoglycemia and hypocalcemia are common, because glucose and calcium are taken up by the mammary gland [6]. This programmed metabolic shift is an example of homeorhesis, namely, the “coordinated changes in metabolism of body tissues necessary to support a physiological state” [7]. Homeorhetic changes occurring during TP cause alterations in circulating metabolites, hormones, and neuroendocrine factors which sustain and drive variations in tissue functions and remodeling, and metabolic flux [6,8].

Mammary gland tissue remodeling facilitates the entry of microorganisms and thereby actives the innate immune system, attracting neutrophils, macrophages, and dendritic cells [9,10]. Indeed, dairy cows around parturition are more susceptible to mastitis [11]. In turn, immune cells produce high levels of pro-inflammatory cytokines such as interleukine-1β (IL-1β), interleukine-6 (IL-6), and tumor necrosis factor-α (TNF-α) [9,10]. A pro-inflammatory state is another difficulty faced by dairy cows during TP, and is well documented in several other species, such as pig [12,13], mice [14], and human [15].

Inflammation is an evolutionarily conserved biological process occurring in response to pathological stimuli, such as tissue injury or infection. It serves in the clearance of pathogens and the resolution of infection, but can also cause deleterious effects on animals’ health and production when excessive or out of control.

Several studies have experimented with the use of nonsteroidal anti-inflammatory drugs (NSAID) during the transition period of dairy cows in order to control the inflammation state. Bertoni et al. [16] observed that the administration of salicylic acid immediately after calving improved, during the first two months, milk production and first-service pregnancy rate. Another NSAID, carprofen, administered for 3 to 4 weeks after calving increased conception rates, but did not affect milk production [17].

The presence of an inflammatory state in postpartum dairy cows is particularly well established. Interestingly, many authors have demonstrated that inflammatory markers and acute-phase proteins increase in cows immediately after parturition, even in apparently healthy animals [18,19,20,21,22], suggesting that parturition and lactogenesis could directly induce a pro-inflammatory status. An alternative to this scenario is that bacterial infections and related endotoxins could affect far more postpartum cows than is currently recognized. It is well established that inflammation is associated with common peripartum diseases. For example, cows affected by fatty liver have elevated plasma concentrations of haptoglobin and serum amyloid A, acute-phase proteins [23] and proinflammatory cytokine TNF-α activity [24]. The difficulty is how to interpret the inflammation–disease association. Indeed, inflammation can contribute to the development of some peripartum disorders (disease cause), but it also possible that subclinical and undiagnosed conditions provoke an elevation of inflammation mediators before diagnosis (disease effect) [25].

As suggested by observational and controlled studies, an important negative consequence of inflammation in dairy cows is a substantial impairment of milk production. Bionaz et al. [19] reported that transition dairy cows with high paraoxonase levels, a negative acute-phase protein, produced about 24% more milk for lactation compared to transition dairy cows with lower paraoxonase levels. Conversely, Huzzey et al. [26] observed that cows with higher concentrations of haptoglobin, a positive acute-phase protein, produced less milk. The administration of TNF-α to dairy cows during the first week of lactation causes a decrease of about 15% of milk production [27]. Hence, inflammation shows deleterious effects on dairy cows’ health in TP when it is excessive and not controlled. There is some evidence that proves that a certain degree of inflammation is necessary to sustain physiological adaptations in TP.

Parturition is facilitated by inflammation; for example, prostaglandin F_2α_ production is mediated by cyclooxygenases, and increases oxytocin receptor expression in the uterus, allowing oxytocin-induced uterine contractions to occur [28]. However, the administration of nonsteroidal anti-inflammatory drugs (NSAID) like aspirin or flunixin meglumine can increase the risk of retained placenta [25].

Milk production could benefit from a low degree of inflammation. It has been speculated that insulin resistance, the key mechanism that diverts nutrient flux from adipose tissue and muscle to the mammary glands, is probably promoted by a certain degree of inflammation [25].

Finally, as mentioned above, inflammation helps dairy cows to fight infections that could occur in early lactation [29].

## 2. The Use of NSAIDs during Transition Period of Dairy Cows

In light of the evidence provided above, inflammation becomes a serious problem for the health of dairy cows in TP when it is excessive and out of control. Numerous studies have investigated the use of several nonsteroidal drugs (NSAID) in the TP of dairy cows to positively affect milk production and the health of cows by controlling the inflammation status [25,30,31,32,33].

NSAIDs act on several prostaglandin pathways. It is known that while cyclooxygenase (COX)-1 acts mainly on daily physiological functions, the function of COX-2 normally acts in specific conditions such as inflammation [34]. Therefore, the therapeutic action of NSAID drugs derives from their ability to inhibit COX-2, while most of the side effects (gastrointestinal irritation, renal toxicity, and inhibition of blood clotting) associated with the use of NSAID are caused by the inhibition of COX-1 [35]. Each NSAID drug acts differently on COX-1 and COX-2. For example, Flunixin meglumine (FM) acts as an inhibitor of both COX-1 and COX-2, with greater selectivity towards COX-1 in horses [36]. Ketoprofen also inhibits both COX-1 and COX-2, showing greater activity as a COX-1 inhibitor [37]. Although both ketoprofen and FM have a prevalent activity on COX-1, the side effects caused by Ketoprofen are minor, due to the short period of time in which ketoprofen remains in circulation compared to FM.

Meloxicam is known to be a preferential COX-2 inhibitor [36], having a targeted action against inflammatory processes and reducing the possibility of experiencing serious side effects, such as placental retention [38].

This review summarizes current knowledge about the effects of NSAIDs on the TP of dairy cows, with regards to the animals’ health status and production. A special section is dedicated to a nonconventional drug utilized in transition dairy cows to improve immune defenses, i.e., pegbovigrastim, a granulocyte colony-stimulating factor.

### 2.1. Salicylates

NSAID drugs have been used in the veterinary field since 1875; the first such compound was salicylic acid (SS), a phenolic molecule extracted from willow bark. SS acts by inhibiting COX-1 and -2 activity [39] through transcriptional factor NF-κB inhibition [40]. The plasma half-life of SS in cows is approximately 30 min [25], so a milk withdrawal time of 24 h is considered adequate to avoid residues contaminating the milk [41] (Table 1).

In recent years, an increasing number of studies on transition period dairy cows have investigated the effects of SS administration on health status and milk production. Bertoni et al. [16] conducted the first study that demonstrated milk yield increment and a reduction of plasma acute phase protein levels, e.g., haptoglobin and ceruloplasmin, in multiparous cows treated in the first 5 days of lactation with lysine acetyl salicylate.

Subsequent studies have confirmed the results of Bertoni’s study by further explaining how the effects of SS on milk production are influenced by both parity and the basal state of inflammation [31,42,43,44]. Farney et al. [42] enrolled in their study both primiparous and pluriparous cows to which SS (≈123 g/d) was administered in water for 7 days. The SS treatment accelerated the early lactation increase in milk fat secretion, leading to an exacerbated negative energy balance as demonstrate by NEFA and BHBA increment and glucose decrement, according to Bertoni et al. (2004), but not milk yield. In a more detailed study, Farney et al. [43] observed a 21% and 14% increment of milk yield and milk total proteins per lactation in SS-treated, third lactation cows; such a phenomenon had not been described previously [42]. The explanation for this apparent discrepancy, even though experimental conditions were similar, may be the fact that authors in the first study [42] did not classify cows according to parity, thereby likely losing the possibility of observing differences in milk yield, as reported also by Bertoni et al. [16], and increased milk protein yield. Some evidence indicates that multiparity increases inflammatory pressure [45], so it is possible to hypothesize that SS treatment could promote milk production (see Table 2 for more details on the experimental methods).

A certain degree of inflammation is necessary to support physiological adaptations in the transition period, but an excess has deleterious effects on milk production. Evidence supporting this hypothesis is provided by the observation of how the presence or absence of an increment in milk yield, milk composition, or milk component yields associated with SS treatment is associated with a different degree of inflammation that cattle experienced at calving, measured as mean haptoglobin concentrations. In one study [44], haptoglobin concentrations were much less than those measured at similar time points in the previous study [31]. Both studies followed the same experimental scheme but the results of milk yield and milk component yields were opposite: in cows with lower haptoglobin concentrations, no differences were observed, whereas in cows with higher haptoglobin concentrations, sustained increases in milk yield were observed in SS cows with respect control cows. A possible adverse effect of the administration of SS is the enhanced risk of the onset of metritis: 30% in SS treated cows compared to 6% of control cows [43]. This data confirms what was previously reported by Bertoni et al. [16]. This increased risk of metritis is likely a consequence of suppressed inflammatory signaling in the immune system. Indeed, the inflammation pathway is essential to activate and attract leukocytes, especially neutrophils, that are critical for the rapid clearance of pathogens [46].

### 2.2. Flunixin Meglumine

The N-methyl-d-glucamine salt of flunixin (flunixin meglumine, FM) is a NSAID drug licensed for use in cattle for the modulation of inflammation in endotoxemia and the control of pyrexia associated with tissue trauma, bovine respiratory disease, and acute bovine mastitis [47]. FM works by inhibiting both cyclooxygenase isoforms COX-1 and COX-2, but is more selective for COX-1 [35].

The terminal half-life of this NSAID is from 3.14 to 8.12 h after slow intravenous administration in cattle [48], and the withdrawal time for milk is 36 h [49] (Table 1). Some studies have reported serious side effects following the use of FM on the day of calving due to an increased risk of placental retention (RP) and metritis [38,50]. In particular, Newby et al. [38] published a large-scale study in which cows were randomly assigned to a group undergoing FM treatment and a negative control group. The authors found that cows treated with FM showed a greater chance of having a high temperature, generally associated with mastitis or metritis. Moreover, the authors highlighted that the administration of FM increased the risk of placental retention and stillbirth and reduced milk production (during the first 14 DIM), but did not influence dry matter intake (DMI) [38]. These two side effects seem to be related to antiprostaglandinic effects which may be specific to this NSAID. Indeed, FM preferentially inhibits COX-1 activity, which is mainly responsible for physiological functions, thus explaining the aforementioned undesirable side effects. In another study, Shwartz et al. [51] observed that periparturient cows subjected to FM treatment did not show improvement of milk production during the first 35 days in milk (DIM), and did not differ in milk fat, protein, and lactose when compared to untreated controls. The FM treatment induced an overall reduction in DMI and caused an increase in rectal temperature in the 2 days after calving [51]. Furthermore, FM treated cows show similar concentrations of plasma glucose and NEFA but significantly lower concentrations of serum nitrogen, probably due to reduced DMI in FM-treated cows [51].

Presumably, the contradictory results between the study of Newby et al. [38] and Shwartz et al. [51] could be ascribed to different FM administration schemes (see Table 2) and to different observation windows, up to 35 DIM in the work of Shwartz et al. [51]. A recent study by Giammarco et al. [52] evaluated the effects caused by a single administration of FM (intramuscular) within 12 h after calving on biochemical parameters, DMI, production performance and the fertility of dairy cows. This study, similar to that of Shwartz et al. [51], confirmed that milk yield and composition were not influenced by single dose FM treatment and, more importantly, that the incidence of retained placenta (RP) was drastically reduced with respect control cows, in contrast to what has been reported by other authors [38,51]. Moreover, as also reported by Shwartz et al. [51], DMI was not influenced by FM treatment. In addition, unlike previous studies, the authors did not highlight changes in body temperature. The latter results indicate that the rise in body temperature and increased risk of RP, i.e., side effects of FM, as described in other studies [38,50], could be FM dose related.

In addition, this study revealed significant differences regarding the culling rate in the period preceding 150 DIM in cows treated with FM compared to control cows (15% vs. 25%), suggesting that treatment with FM may be beneficial to periparturient cows when administered in a single dose. In another study, FM administration was associated with the use of combinations of broad-spectrum antibiotics (long-acting oxytetracycline and sulfadoxyne-trimethoprim) for the treatment of animals suffering with puerpueral metritis, causing a significant reduction in pyrexia and a faster clinical improvement followed by a faster uterine involution and a return to estrus [53].

In contrast, in the study of Drillich et al. [54], the association of a single administration of FM with systemic antibiotic treatment (Ceftiofur, a third-generation cephalosporin) in cows with acute puerperal metritis showed no beneficial effect regarding overall health or reproductive performance.

Overall, all of these studies concurred that FM therapy on the day of calving and immediately afterward should not be recommended due to the aforementioned side effects.

### 2.3. Meloxicam

Unlike FM, meloxicam (MEL) is predominantly a COX-2 inhibitor in horses, rats, humans, dogs, and cats [36,55]; however, its activity has not been well established in cattle. One of the characteristics of MEL is that it has a high oral bioavailability [56] and a mean plasma half-life of approximately 26 h in cattle [57]. If MEL is administered via injection at 0.5 mg/kg of body weight (BW), the milk withdrawal time is 5 days [57], whereas the oral use of MEL at 1 mg/kg of BW has undetectable residues in milk after 80 h [58] (Table 1). It has been shown that cows treated parenterally with a single dose of MEL (0.5 mg/kg of BW) 24 h after calving spend more time at the feed bunk, calculated as a combination of feeding visits and total feeding time; this suggests that the administration of this NSAID could reduce postpartum pain [59]. However, no effects on DMI, milk production, rectal temperature, or plasma NEFA and BHB levels after MEL administration were observed [59]. The same result was reported by another study in which MEL was administered parenterally at the same dose (0.5 mg/kg of BW) as in the previous work [59] but within 6 h after parturition [32]. In contrast to other NSAIDs, the administration of a single dose (0.5 mg/kg of BW) of MEL to cows immediately after calving (within 1 h) did not show a higher incidence of fetal membrane retention or postpartum metritis compared to untreated animals [60]. This is most likely due to the strong affinity of MEL for COX-2 rather than COX-1 enzymes [61]. Two studies, one conducted on a small scale [31] and the other on a large scale [30], provide evidence for how dose and administration mode of meloxicam may influence milk production in opposite ways.

Carpenter et al. [31] enrolled in their study cows from 12 to 36 h postpartum and treated them with MEL as bolus at a dose of 1 mg/kg of BW for three consecutive days. In this study, in contrast to those of Mainau et al. [32] and Newby et al. [59,60], cows treated with MEL showed a clear increase in daily milk production that additionally resulted in a higher milk yield per lactation. Another beneficial effect of treatment with oral MEL of periparturient cows is their minor tendency to leave the herd over the first 365 days post enrolment [31].

In support of what was previously highlighted by Carpenter et al. [31] on the effectiveness of MEL on milk production, there was a large study carried out by Shock et al. [30] on 2673 cows from 20 herds in Canada. Shock et al. [30] highlighted the positive response of milk production over 120 days in lactation following the administration of this NSAID, and also observed a reduction in the onset of subclinical mastitis. In another study [62], it was found that treatment with MEL resulted in a reduced somatic cell count compared to infected cows treated only with the antibiotic [62]. Shock et al. [30] hypothesized that the anti COX-2 action of meloxicam reduces the deleterious effects of the uncontrolled inflammation that occurs postpartum and which induces an increase in acute phase proteins (e.g., haptoglobin and amyloid serum A), thereby facilitating improved immunological responses to infections [63,64,65]. Nevertheless, Carpenter et al. [31] observed that the decrease of somatic cell scores in the first several months of lactation did not correspond to a significant reduction of culling incidence linked to mastitis. Presumably, the significant difference in the numbers of cows enrolled in the trials could differently impact the final results.

Furthermore, treatment with a single oral dose of MEL was associated with a significant reduction in the risk of death within the first 60 days after calving [30,62]. The differences in the results obtained among the various studies could be attributed to the different modes of NSAID administration (orally vs. parenterally), different dosages (0.5 mg/kg vs. 1.0 mg/Kg of BW), and different sample sizes (from a minimum of 30 to 2673 cows) [30,31,32,59,60]. Last but not least, an important confounding factor of NSAID trials is the duration of milk production measurement and health monitoring, varying from 1 month to the whole lactation period, making difficult to compare studies. Overall, meloxicam treatment has been shown to have potential beneficial effects on milk production and cow health when administered very early after calving (within 1 h) without particular side effects.

### 2.4. Carprofen

Carprofen (CA) [(±)-6-chloro-α-methylcarbazole-2-acetic acid], a derivative of propionic acid, is a powerful NSAID that is well tolerated in cattle [66]. It has been found that the administration of CA to cattle has the same degree of effectiveness of FM in inhibiting COX activity, but with a higher selectivity for the COX-2 isoform. For this reason, CA appears to have a better safety margin compared to FM when used in clinical settings [67]. When CA is administered in healthy cows, this NSAID has a small distribution volume (0.09 L/kg), relatively low systemic clearance (2.4 mL/h per kg), and a long terminal half-life (30.7 h). Moreover, when there is an endotoxin-induced mastitis episode, the systemic clearance of CA decreases and its terminal half-life increases (43.0 h) [68].

Vangroenweghe et al. [69] observed that a single dose (1.4 mg/kg) of CA, administered as the first clinical signs worsened, in addition to antibiotic therapy with trimethoprim sulfonamide, in primiparous cows with *E. coli* mastitis had modest modulatory effects on some inflammatory markers but evident effects on some of the main clinical markers, such as rectal temperature (RT) and reticulorumenic motility. There were evident beneficial effects regarding the general clinical conditions, recovery of the milk composition, and reduction of the production of eicosanoids [69].

Priest et al. [17] noted that, in contrast to what they had hypothesized, the administration of CA between 21 and 31 DIM in cows suffering from subclinical endometritis increased neither the portion of uterine PMN at 42 DIM nor milk production, but increased rates of pregnancy in cows with high PMN and improved liver function in all cows. Assuming that the lack of effect of CA on milk production reported by Priest et al. [17] was due to too late administration of the drug, Meier et al. [33] carried out a large-scale study in which a group of 639 cows received treatment on days 1, 3, and 5, and another group on days 19, 21, and 23 after calving. However, no benefits on milk production, metabolic health indicators, or most reproductive measures were found up to 6 weeks after calving.

The aforementioned results were confirmed in a study of Giammarco et al. [52], where CA treatment did not affect milk yield or quality, dry matter intake, BW, or body condition score. Furthermore, the authors reported that CA treatment significantly affected culling rate, reducing it in the period before 150 DIM compared with control cows, but that it is less effective than FM treatment in the reduction of RP risk. In addition, cows treated with CA also showed a higher percentage of pregnancy at first insemination than untreated cows [52].

In contrast to the other studies, Stilwell et al. [70] found that the early administration of CA (within 6 h after calving) induced higher milk production, which began at 220 DIM, becoming significantly higher at 305 DIM in primiparous cows compared to untreated animals. Compared to untreated cows, a large number of CA-treated cows ate during the postpartum period (21% vs. 0%), probably because CA reduces pain-associated inappetence [71,72] and potentially lowers the negative energy balance that commonly occurs in early lactation. However, the number of animals returning to pregnancy after calving at 220 days was greater in the control group [70], probably because higher milk production delays the resumption of ovarian function [73].

Overall, although studies about the effects of CA treatment on periparturient dairy cows are scarce, those that exist suggest that this NSAID positively influences the culling risk and pregnancy at first insemination, whereas its effect on milk production is probably associated with administration timing and is limited to primiparous cows.

## 3. Another Drug: Pegbovigrastim

Different management strategies have been studied to reduce the negative effects on periparturient dairy cows’ health of the dramatic metabolic changes which occur in the first phase of lactation. In recent years, a new formulation was launched in the EU and US markets: the immune-stimulant Pegbovigrastim. Pegbovigrastim is a long-acting analogue of recombinant bovine granulocyte colony-stimulating factor (bG-CSF) that increases the count and functionality of polymorphonuclear cells in dairy cows. 

A phenomenon occurring during the transition period of dairy cows is the impairment of the function of neutrophils [74]. Several studies have shown that in cattle, the administration of Pegbovigrastim provides a well-tolerated approach to overcoming periparturient immune suppression, making it possible to restore normal neutrophil function [62,75]. The administration of Pegbovigrastim induces a state of neutrophilia, characterized by a ‘left-shift’ towards progenitor cells with a release of mature neutrophils and band cells from storage pools in bone marrow [76,77]. Pegbovigrastim is found to induce a slight decrease of red cell count and hematocrit in cows treated 7 and 21 days after calving [62,77]. The finding of normocytic, hypochromic anemia in 50% (8/16) and 19% (3/16) of the Pegbovigrastim treated and control cows, respectively, could indicate an increased anemic effect after treatment with this immune stimulant [77]. A relevant effect of Pegbovigrastim is its ability to enhance the transcription of cell adhesion, migration, recognition, antimicrobial activity, and inflammation cascade genes by leukocytes, which, in turn, could trigger immune cell activation, thereby improving their function [78].

There is little information about the effects of Pegbovigrastim on postpartum disease occurrence and production. A recent paper [79] analyzed the effects of Pegbovigrastim in transition dairy cows, showing a 25% greater incidence of clinical metritis and a 2.46-fold increase in the likelihood of developing the disease in treated cows compared to controls. The increase in clinical metritis occurrence in this study agrees with data reported by Ruiz et al. [80], who observed that the administration of Pegbovigrastim resulted in greater incidence of metritis (17.6%) and greater odds (16.4%) of developing metritis in the first 21 days after calving. Clinical mastitis and puerperal metritis occurrence did not differ between treatments [79]. Moreover, no significant effects were registered on milk yield, fat, protein, lactose, nonfat solids [5], or somatic cells count in Pegbovigrastim-treated cows [5,79].

## 4. Conclusions

It is now clear that most dairy cows experience a subacute inflammation status for the first days after calving that is physiological, to a certain degree, as it is important for normal immunity and reproductive system function, and for homeorhetic adaptations. If the degree of inflammation exceeds a certain level, it becomes harmful, impairing lactation. In such cases, the use of NSAIDs could be beneficial. The potential benefits of the treatment of periparturient cows with NSAIDs depend by the type of drug employed and its dosage and administration mode. NSAIDs that preferentially inhibit COX-1 activity, such as salicylates and Flunexin meglumine, show important side effects like increased risk of retained placenta, culling, or metritis, even if milk production is, on average, improved. In the case of salicylates, cow inflammation levels seem to be important; in fact, in cows with high haptoglobin concentrations, the administration of salicylate positively influences milk production compared to what occurs in cows with lower haptoglobin concentrations [31,44].

Positive effects of NSAIDs, that preferentially inhibit COX-2 activity, on milk production seem to be plausible. Their influence on cows’ health is well recognized, with a minor culling and death risk and mastitis incidence and with an increase of pregnancy at first insemination.

At present, there are no irrefutable studies demonstrating the safety and efficacy of NSAIDs. Therefore, their routine use to improve milk production and dairy cow health should be carefully evaluated and possibly associated with early diagnoses of high inflammation status.

## Figures and Tables

**Table 1:** Principal qualitative aspects of nonsteroidal anti-inflammatory drugs (NSAIDs).

Drug	Target	Half Time	Milk Withdrawal
Salicylate	COX-1/2	0.5 h	24 h
Flunexin meglumine	COX-1 preferential	1.6 h	36–48 h
Meloxicam	COX-2 preferential	26 h	120 h (0.5 mg/kg BW, i.m.) or 80 h (1 mg/kg BW, os)
Carprofen	COX-2 selective	30.7 h	Not required ^1^

^1^ in European countries; i.m.: intramuscular; os: oral.

**Table 2:** Summary of studies in periparturient dairy cows reporting doses and timing of treatments with commonly-used nonsteroidal anti-inflammatory drugs (NSAIDs) investigated during the periparturient period of dairy cows.

NSAIDs	N° Total Cows	Dose	Timing ^1^	N° Treatment Days	References
Salicylates	78	1.95 g/L (os)	DIM 1 to 7	7	[42]
	78	1.95 g/L (os)	DIM 1 to 7	7	[43]
	51	185 mg/kg (os)	12 to 36 h ^2^	3	[31]
	56	125 g/d (os)	12 to 36 h ^2^	3	[44]
	22	15 g/d + 7.5 g/d (i.m.)	DIM 1 to 3 + DIM 4 to 5	5	[16]
Flunexin meglumin	26	2.2 mg/kg (i.v.)	within 5 h ^2^	3	[51]
	1265	1.1 to 2.2 mg/kg (i.v)	within 24 h ^2^	2	[38]
	49	1–5 g in 30 mL (i.m.)	immediately after caesarean	1	[50]
	60	2.2 mg/kg (i.m)	within 12 h ^2^	1	[52]
	128	2.2 mg/kg (i.v.)	DIM 5 to 8 ^2^	2 bid + 2 sid	[53]
	119	2.2 mg/kg (i.v)	DIM 4 to 5 ^2^	1	[54]
Meloxicam	103	0.5 mg/kg (s.c.)	25.4 h ^2^	1	[59]
	462	0.5 mg/kg (s.c.)	within 1 h ^2^	1	[60]
	60	0.5 mg/kg (s.c.)	within 6 h ^2^	1	[32]
	51	~1 mg/kg (os)	12–36 h ^2^	4	[31]
	1.009	~1 mg/kg (os)	at calving	1	[30]
	361	0.5 mg/kg (s.c.)	at the diagnosis of c.m.	1	[62]
Carprofen	639	1.4 mg/kg (s.c.)	DIM: 1, 3, 5; DIM 19, 21, 23	3	[33]
	213	1.4 mg/kg (s.c.)	DIM 21 (every 3 days)	3	[17]
	39	1.4 mg/kg (i.v.)	immediately after calving	1	[70]
	60	1.4 mg/kg (s.c.)	within 12 h ^2^	1	[52]

^1^ start treatment; ^2^ hours after calving; DIM: Day in Milk; c.m.: clinical mastitis; bid: *bis in die*, twice a day; sid: *semel in die*, once a day; i.m.: intramuscle; i.v.: intravenous: s.c.: subcutaneous; os: oral.
